# Mapping Depression in Schizophrenia: A Functional Magnetic Resonance Imaging Study

**DOI:** 10.1093/schbul/sbv186

**Published:** 2015-12-27

**Authors:** Veena Kumari, Emmanuelle Peters, Ashley Guinn, Dominic Fannon, Tamara Russell, Alexander Sumich, Elizabeth Kuipers, Steven C. R. Williams, Dominic H. ffytche

**Affiliations:** ^1^Department of Psychology, King’s College London, Institute of Psychiatry, Psychology and Neuroscience, London, UK;; ^2^NIHR Biomedical Research Centre for Mental Health, South London and Maudsley NHS Foundation Trust, London, UK;; ^3^Department of Old Age Psychiatry and Department of Neuroimaging Sciences, King’s College London, Institute of Psychiatry, Psychology and Neuroscience, London, UK;; ^4^Department of Psychosis Studies, King’s College London, Institute of Psychiatry, Psychology and Neuroscience, London, UK;; ^5^Department of Neuroimaging, King’s College London, Institute of Psychiatry, Psychology and Neuroscience, London, UK

**Keywords:** psychosis, depressive symptoms, fMRI, fear, thalamus

## Abstract

Depressive symptoms are common in schizophrenia, often left untreated, and associated with a high relapse rate, suicidal ideation, increased mortality, reduced social adjustment and poor quality of life. The neural mechanisms underlying depression in psychosis are poorly understood. Given reports of altered brain response to negative facial affect in depressive disorders, we examined brain response to emotive facial expressions in relation to levels of depression in people with psychosis. Seventy outpatients (final *N* = 63) and 20 healthy participants underwent functional magnetic resonance imaging during an implicit affect processing task involving presentation of facial expressions of fear, anger, happiness as well as neutral expressions and a (no face) control condition. All patients completed Beck Depression Inventory (BDI-II) and had their symptoms assessed on the Positive and Negative Syndrome Scale (PANSS). In patients, depression (BDI-II) scores associated positively with activation of the left thalamus, extending to the putamen-globus pallidus, insula, inferior-middle frontal and para-post-pre-central gyri during fearful expressions. Furthermore, patients with moderate-to-severe depression had significantly higher activity in these brain regions during fearful expressions relative to patients with no, minimal, or mild depression and healthy participants. The study provides first evidence of enhanced brain response to fearful facial expressions, which signal an uncertain source of threat in the environment, in patients with psychosis and a high level of self-reported depression.

## Introduction

Depression has been regarded as a key feature of schizophrenia since it was first defined by Bleuler.^1^ A large body of evidence confirms that depressive symptoms are a core component,^2,3^ and may precede the clinical manifestation^4,5^ of psychosis. Prevalence rates for major depressive disorder (MDD) comorbid with psychotic disorders commonly vary between 25% to 75%,^6,7^ and around 70% of chronic schizophrenia patients without a MDD may show signs of sub-syndromal depression.^2,3^ Although there is overlap between depressive and negative symptoms, a distinction between them can be made,^8,9^ with negative symptoms indicating the severity of illness and depressive symptoms reflecting motivational hopelessness about the future.^8^ Importantly, depressed mood in schizophrenia is known to have detrimental consequences, such as a high relapse rate, suicidal ideation, increased mortality, reduced social adjustment and poor quality of life.^8,10–14^ A key question, central to refining and developing effective treatments for depression in schizophrenia is: what are the psychobiological abnormalities associated with depressive symptoms in schizophrenia, and to what extent are they similar or different to those commonly observed in MDD?

A large number of functional magnetic resonance imaging (fMRI) studies have already examined the neurobiology of MDD, particularly using facial affect processing paradigms.^15^ MDD patients, relative to healthy participants, show hyper-activation to negative facial affect, in particular sadness and fear, in the amygdala, insula, parahippocampal gyrus, fusiform face area and putamen.^16–19^ Structural and functional alterations in these regions are widely implicated in disturbed implicit/explicit processing of emotive stimuli and poor affect regulation in MDD.^20–22^ Schizophrenia patients, on the other hand, are reported to show, on average, reduced activation of the amygdala, parahippocampal gyrus, superior frontal gyrus, insula and basal ganglia to negative facial affect.^23,24^ There are no fMRI studies to our knowledge specifically examining the role of depression in brain responses to negative facial affect in schizophrenia. Amygdala activation to fearful faces, however, was found to correlate positively with the severity of flat affect, a negative symptom dimension, in a previous study.^25^

There is indirect support from structural brain imaging and neuropsychological studies that depressed mood in schizophrenia and MDD could have at least partially overlapping neurobiology, such as volumetric abnormalities of the temporal lobe and anterior cingulate^26^ and attentional impairment.^27^ Depression in schizophrenia, however, differs markedly amongst patients depending on the stage of the illness, with more severe depressive symptoms in first episode patients, perhaps as a natural reaction to developing psychotic symptoms, relative to multi-episode chronic patients.^28,29^ Depression in later phases of the illness is found to be more subjective and best detected with self-report instruments,^29,30^ such as the Beck Depression Inventory.^31^

In this study, our aim was to elucidate the neural correlates of subjective depression, using whole brain fMRI during an established facial affect paradigm,^32^ in patients with schizophrenia or schizoaffective disorder. Assuming depression in chronic psychosis patients to share functional brain abnormalities associated with MDD,^15–17^ we expected to find a positive association between the level of self-reported depression and brain activity, particularly in the amygdala, insula, parahippocampal gyrus, fusiform face area and putamen, during negative facial affect processing. In addition, we studied a group of healthy participants to examine whether a positive association between depression and brain activity in patients, if found, reflected a hyperresponse, or a stronger response within the normal range, in patients with moderate-to-severe depression.

## Methods and Materials

### Participants and Design

The study involved 70 right-handed^33^ outpatients with a DSM-IV diagnosis^34^ of schizophrenia or schizoaffective disorder, recruited from the South London and Maudsley NHS Foundation Trust (SLaM), United Kingdom. Twenty right-handed^33^ healthy participants (with no current or previous diagnosis of an Axis I disorder^35^) matched, on average, to age and sex of the patient group were also studied.

All recruited patients met the following inclusion criteria: (1) aged 18–65 years, (2) on a stable dosage of antipsychotic medication for at least 3 months prior to taking part, (3) no history of neurological impairments, systematic illness or head injury, (4) no current substance use disorder, and (5) able to provide written informed consent. The final study sample consisted of 63 patients (table 1), out of 70 recruited initially, for whom we had obtained usable fMRI data, symptom ratings using the Positive and Negative Syndrome Scale (PANSS)^36^ and who had fully completed (within 1wk of fMRI) the Beck Depression Inventory-II (BDI-II).^31^ PANSS assessments on patients were carried out by an experienced psychiatrist (D.F.) who was blind to patients’ BDI-II scores.

**Table 1.** Demographic and Clinical Characteristics and Task Performance of Study Sample

Demographic and Clinical Measures		Patients					Healthy Participants (*n* = 20;75% Men)	
		All Patients (*N* = 63; 56 With Schizophrenia and 7 With Schizoaffective Disorder; 73% Men)		No/Minimal Depression (*N* = 25; 22 Schizophrenia, 3 Schizoaffective Disorder; 80% Men)	Mild Depression (*N* = 17; 16 Schizophrenia, 1 Schizoaffective Disorder; 70.6% Men)	Moderate-to-Severe Depression (*N* = 21; 17 Schizophrenia, 4 Schizoaffective Disorder; 66.7% Men)		
		Mean (SD)	Range	Mean (SD)	Mean (SD)	Mean (SD)	Mean (SD)	Range
Age		38.19 (9.77)	19–61	39.36 (9.82)	34.76 (8.23)	39.57 (10.61)	37.25 (12.49)	20–65
Years in education		13.87 (2.58)	8–20	14.32 (2.72)	12.94 (2.70)	14.09 (2.23)	14.80 (2.98)	10–20
Predicted IQ (^a^NART)^d^		107.94 (9.62)	86–124	108.17 (10.08)	108.70 (10.29)	107.09 (8.92)	115.74 (7.46)	91–126
^b^BDI-II		16.79 (11.68)	0–54	5.80 (4.07)	16.12 (1.49)	30.43 (7.38)	2.80 (2.91)	0–7
Age at illness onset (y)		25.59 (8.58)	15–48	24.64 (8.15)	25.12 (6.10)	24.57 (9.81)	n/a	
Duration of illness (y)		13.46 (9.56)	1–43	14.72 (8.32)	9.71 (7.27)	15.00 (11.80)	n/a	
^c^Positive symptoms		16.08 (4.75)	7–25	15.80 (4.71)	16.64 (5.19)	19.95 (4.62)	n/a	
^c^Negative symptoms		17.65 (4.82)	7–27	16.52 (4.81)	18.94 (4.74)	17.95 (4.83)	n/a	
^c^General psychopathology		32.22 (6.62)	18–56	29.76 (6.37)	34.18 (7.68)	33.57 (5.24)	n/a	
^c^PANSS: total symptoms		65.95 (13.48)	37–108	62.08 (13.26)	69.76 (15.68)	67.48 (11.03)	n/a	
Antipsychotic medication (mg) (in chlorpromazine equivalents)^e^		449.51 (295.21)	100–1600	430.80 (215.51)	495.58 (10.29)	433.50 (327.01)	n/a	
		*N* (%)		*N* (%)	*N* (%)	*N* (%)	*N* (%)	
Antidepressant medication		22 (34.9%)		7 (28%), 2 on Fluoxetine, 2 Citalopram, 1 Sertraline, 1 Mirtazipine, 1 Clomipramine	7 (41%), 2 on Fluoxetine, 1 Citalopram, 2 Venlafaxine, 1 Paroxetine, 1 Clomipramine	8 (38%), 2 on Fluoxetine, 2 Citalopram, 1 Venlafaxine, 1 Mirtazapine, 1 Amitriptyline	0 (0%)	
Cigarette smoking		34 (54%)		13 (52%)	9 (52.9%)	13 (57.1%)	7 (35%)	
Task performance		All patients		Low depression	Moderate depression	High depression	Healthy participants	
Accuracy for correct gender discrimination responses (%)	Condition	Mean (SD)		Mean (SD)	Mean (SD)	Mean (SD)	Mean (SD)	
	Neutral	89.14 (11.73)		87.32 (11.71)	92.46 (9.18)	88.54 (13.45)	92.50 (18.29)	
	Fearful	88.94 (15.46)		89.00 (10.32)	92.28 (14.07)	86.16 (20.91)	92.66 (15.84)	
	Angry	84.62 (15.79)		81.87 (16.36)	90.44 (12.47)	83.18 (16.92)	91.25 (19.38)	
	Happy	93.06 (9.23)		92.12 (9.42)	95.96 (4.91)	91.82 (11.33)	94.53 (18.66)	
Detection (%)	No face	93.03 (16.16)		94.37 (10.57)	92.09 (15.47)	92.19 (21.91)	93.75 (22.25)	
Reaction time (RT) to correct responses (s)	Neutral	1.05 (0.23)		1.09 (0.24)	1.00 (0.18)	1.05 (0.26)	0.94 (0.32)	
	Fearful	1.04 (0.23)		1.12 (0.27)	0.99 (0.20)	1.01 (0.19)	0.91 (0.33)	
	Angry	1.03 (0.25)		1.10 (0.27)	0.97 (0.22)	1.00 (0.22)	0.95 (0.32)	
	Happy	1.02 (0.24)		1.06 (0.28)	0.97 (0.20)	1.01 (0.22)	0.92 (0.35)	
Detection RT (s)	No face	0.79 (0.24)		0.84 (0.27)	0.79 (0.23)	0.72 (0.20)	0.62 (0.17)	

*Note*: ^a^NART: National Adult Reading Test.^37^

^b^BDI-II: Beck Depression Inventory-II.^28^

^c^PANSS: Positive and Negative Symptom Scale.^32^

^d^Data missing for 1 patient and 1 healthy participant.

^e^Reliable dose information missing for 4 (1 no/minimal, 1 mild and 2 moderate-to-high depression) patients.

The study procedures were approved by the ethics committee of the joint research ethics committee of the SLaM and the Institute of Psychiatry, London. All participants provided written informed consent prior to their participation and were compensated for their time and travel.

### fMRI Paradigm and Procedure

The task involved presentation of black and white pictures of faces from Ekman and Friesen’s set.^37^ Three types of emotions (fear, anger, happy; 100% expression) and relatively neutral expressions were displayed in 30-second blocks of 8 pictures each, with each picture presented for 3.75 seconds. There were sixteen 30-second blocks with facial expressions (4 fear, 4 anger, 4 neutral, 4 happy), and each block was followed by a 15-second control block during which just an oval frame, as on facial expression trials and matched for luminance but without the face inside, appeared 4 times (order of presentation over the 12-minute experiment: neutral-control-fear-control-happy-control-anger-control-fear-control-anger-control-neutral-control-happy-control-anger-control-happy-control-fear-control-neutral-control-happy-control-neutral-control-anger-control-fear-control). The oval frame, rather than neutral expressions, was used as the control condition because it has been observed that people with schizophrenia^38,39^ or those at a high risk for psychosis^40^ may activate brain regions to neutral faces that are normally associated with fear processing. On presentation of each facial expression, participants were required to indicate whether the face was a male/female by pressing the left (female)/right (male) button on a button box, simply to ensure they were attending to the stimuli. During the no face (oval frame) condition, attention was monitored by asking participants to press either button (left/right) when the blank oval frame appeared. All participants underwent task familiarization in advance of the scan through practice of the gender discrimination task once on all identities used in the fMRI experiment on a laptop computer and an identical button box device. The task and administration procedures were identical to that used in a previous study.^32^ Participants were requested to abstain from alcohol for at least 24 hours prior to their scanning. They were provided with tea and decaffeinated coffee, as requested, up until 40 minutes before fMRI. Smokers were allowed to smoke as usual to avoid a state of smoking/nicotine withdrawal but care was taken not to start fMRI scanning them within 40 minutes of smoking a cigarette.

### Image Acquisition

Echoplanar MR brain images were acquired using a 1.5 T GE Signa system (General Electric). In each of 16 near-axial noncontiguous planes parallel to the inter-commissural plane, 240 T2*-weighted MR images depicting blood-oxygen-level-dependent (BOLD) contrast (echo time [TE] 40ms, repetition time [TR] 3s, Flip 90°, field of view 240mm, matrix 64×64, in-plane resolution 3.1mm, slice thickness 7.0mm, interslice gap 0.7mm) were acquired over the 12-minute experiment. In the same session, a high resolution 3-D inversion recovery prepared spoiled GRASS volume dataset was acquired with TE = 5.3ms, TI = 300ms, TR = 12.2ms, in-plane resolution = 0.94mm, slice thickness = 1.5mm.

### Data Analysis

All behavioral analyses were performed using Statistical Package for Social Sciences (version 22). Alpha level for testing significance of effects was maintained at *P* < .05 unless stated otherwise.

#### Demographic, Behavioral and Clinical Measures.

Patients and healthy participants were compared on age, education, predicted IQ^41^ and BDI-II scores using independent sample *t* tests. Patient subgroups with varying depression levels (no/minimal: BDI-II score 0–13; mild: score 14–19, moderate-to-severe: score 20 or above) were compared on age, education, predicted IQ^41^ and PANSS symptoms using (separate) 1-way ANOVA, with Depression (no/minimal, mild, moderate-to-severe) as a between-subjects factor, followed by independent sample *t* tests where needed.

Group and emotion effects in performance accuracy (% correct gender discrimination in facial expressions) and latency (reaction time to correct responses) were analysed separately using a Group (patients, healthy participants) × Emotion type (fear, anger, neutral, happy) repeated-measures ANOVA with Group as the between-subjects factor and Emotion type as the within-subjects factor, followed by post hoc mean comparisons. The effects of varying depression in performance accuracy and latency of patients were analyzed (separately) using Depression × Emotion type ANOVA with Depression as the between-subjects factor and Emotion type as the within-subjects factor, followed by lower order ANOVAs and post hoc mean comparisons. Finally, Pearson’s correlations were used to examine relationship of BDI-II scores to performance (accuracy and latency), symptoms, age, illness duration and the doses of antipsychotic medication in the patient group.

### fMRI

#### Preprocessing.

For each participant, the 240 volume functional time series were realigned to the first volume, corrected for motion artefacts (*x*, *y* and *z* translation, pitch, roll, yaw; time series with translations >5 mm or rotations > 5° were excluded from the analysis), transformed into stereotactic space using the EPI template in SPM (affine transformation x, y and z, 16 nonlinear iterations, 7×9 × 7 basis functions), spatially smoothed with a 10mm FWHM Gaussian filter and band pass filtered using statistical parametric mapping software (SPM5; http://www.fil.ion.ucl.ac.uk/spm).

#### Models and Statistical Inferences.

Data were analyzed using a random effect procedure.^42^ Participant-specific activations were identified with a factorial model consisting of 4 facial expression conditions, and no face control condition serving as an implicit baseline. The boxcar for each 30-second epoch was convolved with the haemodynamic response function. Motion parameters were included as covariates at this stage. The second stage of the random effect model identified generic task-related activations in the patient and healthy groups (separately) for each facial expression condition (fear, anger, neutral, happy) vs no face control condition (height threshold *P* < .005; cluster-corrected *P* ≤ .05), using statistical parametric mapping software (SPM8; http://www.fil.ion.ucl.ac.uk/spm).

The relationship of BDI-II scores in patients with neural activity across the whole brain was first examined (height threshold *P* < .05; cluster-corrected *P* ≤ .05), using regression model within SPM8 with BDI-II scores entered as a covariate for each facial expression condition (fear, anger, neutral, happy) vs no face control condition; and also for each emotional expression (fear, anger, happy) vs the neutral expression following the observation of no relationship between BDI-II scores and activity to neutral expressions (see Results). In addition, all regression analyses were repeated with gender included as an additional covariate. Since the inclusion of gender revealed the same cluster associations, the results of these additional SPM regressions are not reported hereafter. The participant-specific activation values were then extracted from the clusters (peak voxel) that showed a relationship with BDI-II scores in the patient group, and examined (within the SPSS) for their possible relationships with symptoms, age, illness duration and the doses of antipsychotic medication (in chlorpromazine equivalents) using Pearson correlations.

Next, we extracted participant-specific mean BOLD signal for all patients and healthy participants from each of 2 activation clusters (thalamus and superior frontal gyrus [SFG]; 10mm sphere around the peak voxel) that showed a significant association with BDI-II scores during fearful expression in SPM regression analyses (table 2) using the MarsBaR toolbox (http://marsbar.sourceforge.net/projects/marsbar). These data were then analysed (within the SPSS) to confirm the influence of depression in patients’ brain activity using a Region (thalamus, SFG) × Emotion type (fear, anger, happy, neutral) × Depression (no/minimal, mild, moderate-to-severe) ANOVA, followed by lower order ANOVAs and mean comparisons as needed. A further Region (thalamus, SFG) × Group (healthy participants, no/minimal depression patients, mild depression patients, moderate-to-severe depression patients) ANOVA was conducted to establish whether higher activations during fearful expressions in patients with moderate-to-high depression, relative to those with no/minimal/mild depression (detected with the earlier ANOVA), reflected a hyperresponse or a stronger response within the normal range.

**Table 2.** Significant Associations Between Brain Activity to Fearful Expressions and Depression in Patients (Voxel Threshold *P* < .005 Uncorrected)

Fearful > No Face Control								
Brain Area	BA	Voxels (*n*)	Side	MNI Coordinates			Voxel T	Cluster-Corrected *P*
				*x*	*y*	*z*		
Ventral posterior medial nucleus (thalamus)		5422	Left	−16	−22	0	4.31	.018
Postcentral gyrus	3		Left	−50	−14	48	3.17	
Globus pallidus (lentiform nucleus)			Right	18	−12	−4	3.10	
Supramarginal gyrus	40		Left	−32	−50	32	3.06	
Insula			Left	−36	−48	24	3.01	
Inferior frontal gyrus	9		Left	−48	6	28	2.95	
Postcentral gyrus	3		Left	−48	−20	48	2.93	
Precentral gyrus	4		Left	−32	−20	52	2.91	
Paracentral lobule	5		Left	−14	−44	62	2.89	
Postcentral gyrus	2		Left	−48	−24	52	2.86	
Supramarginal gyrus	40		Left	−36	−48	30	2.84	
Postcentral gyrus	2		Left	−48	−24	46	2.73	
Superior parietal lobule	7		Left	−34	−52	50	2.73	
Fearful > Neutral								
Superior frontal gyrus	6	9081	Right	4	10	60	4.49	<.001
	6		Right	4	16	58	4.11	
Medial frontal gyrus	6		Right	14	−4	60	3.94	
	6		Right	12	0	60	3.91	
Middle frontal gyrus	6		Right	42	−4	46	3.51	
Superior frontal gyrus	6		Right	8	22	56	3.50	
Precentral gyrus	6		Left	−38	−10	58	3.28	
Medial frontal gyrus	6		Left	−12	2	62	3.26	
Middle frontal gyrus	6		Left	−20	−20	62	3.24	
Precentral gyrus	4		Left	−32	−20	52	3.24	
	6		Right	50	2	46	3.23	
Precentral gyrus	6		Left	−24	−22	62	3.17	
Superior frontal gyrus	8		Right	10	26	54	3.15	
Precentral gyrus	4		Left	−50	−10	46	3.08	
Anterior cingulate	24		Left	−12	0	52	3.08	
Ventral posterior medial nucleus (thalamus)		3805	Left	−18	−22	2	3.74	.009*
Insula			Left	−40	−12	10	3.30	
Hippocampus			Right	30	−22	−8	2.78	
Mammillary body (thalamus)			Right	10	−18	−2	2.72	
Thalamus			Right	10	−16	4	2.70	
Putamen (lentiform nucleus)			Right	28	−18	8	2.69	
Transverse temporal gyrus	41		Left	−52	−16	8	2.68	
Putamen (lentiform nucleus)			Right	30	−18	4	2.67	
Claustrum			Right	34	−22	2	2.64	
Ventral lateral nucleus (thalamus)			Right	20	−16	8	2.43	
Globus pallidus (lentiform nucleus)			Right	20	−12	0	2.35	
Caudate			Right	10	4	8	2.30	

*Note*: BA, Brodmann area; MNI, Montreal Neurological Institute.

*Uncorrected *P*.

## Results

### Demographic, Behavioral and Clinical Measures

Patients and healthy participants were comparable (table 1) on age, sex distribution and education (*P* values > .18) but patients had significantly lower premorbid IQ (*t*_79_ = 3.21, *P* = .002). Patients also rated themselves much higher on BDI-II depression (*t*_81_ = 5.46, *P* < .001). Patients with varying levels of depression (table 1) did not differ in age, sex distribution, education, age at illness onset, illness duration, antipsychotic dose, and PANSS positive, negative or total symptoms (*P* values > .16) but differed marginally in general psychopathology scores (*F*_2,60_ = 1.90, *P =* .05). The no/minimal depression subgroup, relative to the mild (*F*_1,44_ = 4.79, *P =* .03) and moderate-to-severe (*F*_1,40_ = 4.12, *P =* .05) depression subgroups had lower general psychopathology scores (accounted for by the positive correlation, described below, between the BDI-II and general psychopathology scores).

Both healthy and patient groups showed a high level of gender discrimination accuracy across all conditions (table 1). This was expected given the easy task demands, implemented simply to ensure patients’ attention to task stimuli and not place cognitive demands on them. For accuracy, there was a significant main effect of Emotion type (*F*_3,243_ = 5.84, *P* < .001) but there was no effect of Group (*F*_1,81_ = 1.30, *P* = .26) or a Group × Emotion type interaction (*F*_3,243_ = 1.15, *P =* .33). The main effect of Emotion type indicated a lower accuracy for angry expressions than for fearful (*t*_82_ = 2.36, *P =* .02), neutral (*t*_82_ = 3.21, *P* = .002) and happy expressions (*t*_82_ = 6.32, *P* < .001), and in addition, for fearful (*t*_82_ = 0.89, *P* = .005) and neutral (*t*_82_ = 5.33, *P* < .001), relative to happy expressions. There was no significant difference between fearful and neutral expressions (*t*_82_ = 0.09, *P* = .93). For latency, no significant effects were found (Emotion type: *F*_3,243_ = 1.58 *P* = .19; Group: *F*_1,81_ = 2.83, *P* = .10; Emotion type × Group: *F*_3,243_ = 1.23, *P* = .30).

Patient subgroups with varying depression levels also showed comparable accuracy across all conditions (table 1), with no significant effect of Depression (*F*_2,60_ = 1.42, *P* = .25). There was a significant effect of Emotion type (*F*_3,180_ = 9.14, *P* < .001; reflecting the same pattern as above) but no Depression × Emotion type interaction (*F*_6,180_ = 0.56, *P =* .76). There was no effect of Depression (*F*_2,60_ = 1.38, *P =* .26), or any other effect, in latency.

In patients, BDI-II (depression) scores had modestly positive correlation with PANSS general psychopathology (*r* = .309, *P* = .014; for PANSS-G6 depression item, ρ = .353, *P* < .01) but had no correlation with PANSS positive (*r* = .01) or negative symptoms (*r* = .11). BDI-II scores did not correlate with accuracy or latency during any task condition (all *P* > .10), age (*r* = .02, *P* = .94), illness duration (*r* = .006, *P* = .96) or doses of antipsychotic medication (*r* = .004, *P* = .98).

### fMRI

#### Generic Task Related Activations in Patients and Healthy Participants.

**Fig. 1.**
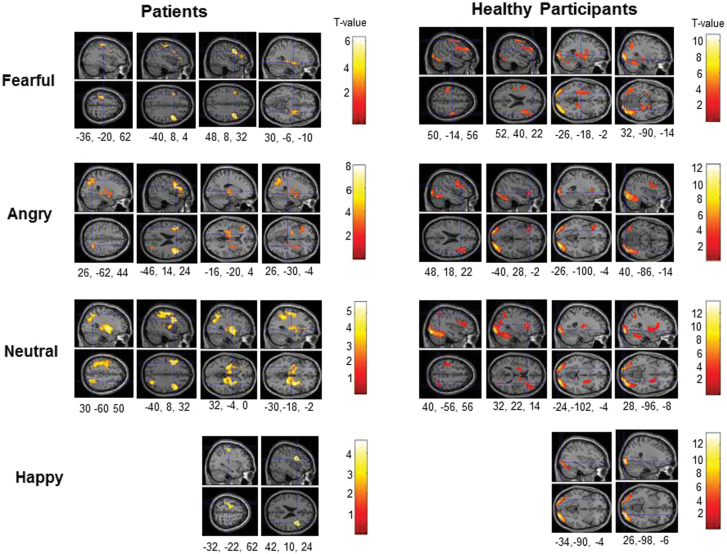
Task related increases (facial expressions > no face control stimuli) in patient and healthy groups with associated Montreal Neurological Institute (MNI) *x*, *y* and *z* coordinates (maps thresholded at *P* = .005 uncorrected). For each coordinate, sagittal and axial slices are shown. The left hemisphere is shown at the top of the axial slices.

The areas showing significant activity changes for each facial expression condition vs no face control condition in patient and healthy groups are described in supplementary appendix 1, and displayed in figure 1 (the clusters which did not reach corrected cluster-level significance are also displayed in figure 1 for completeness).

In patients, robust activation for fearful expressions (>no face) was limited to the right inferior frontal gyrus, with weaker (uncorrected; displayed in figure 1) activity seen in the left inferior frontal gyrus (peak, *x* = −40, *y* = 8, *z* = 32, cluster size = 417 voxels), right striatum-hippocampus-parahippocampal gyrus (*x* = 30, *y* = −6, *z* = −10; 401 voxels), and the left precentral gyrus (*x* = 36, *y* = −20, *z* = 62; 417 voxels; figure 1). Significant activations for angry expressions included the inferior frontal gyrus (bilateral), thalamus (bilateral), parahippocampal gyrus (right), globus pallidus (left) and middle occipital gyrus (right). For neutral expressions, robust activation was seen in the middle frontal gyrus (bilateral), precentral gyrus (left), thalamus (bilateral) and globus pallidus (left); in addition, there was weak (uncorrected level) activation of the left superior parietal cortex (*x* = 30, *y* = −60, *z* = 52; 721 voxels; figure 1). No area was activated for happy expressions at the corrected level but 2 clusters with uncorrected significance were present, 1 in the right inferior-middle frontal gyrus (*x* = 42, *y* = 10, *z* = 24; 447 voxels) and the other in the left precentral gyrus (*x* = −32, *y* = −22, *z* = 62; 461 voxels; figure 1).

In healthy participants, fearful expressions activated parts of the inferior occipital gyrus, inferior-middle frontal gyri, striatum, insula, amygdala, hippocampus, parahippocampal gyrus and the thalamus. Angry expressions mainly activated the inferior occipital gyrus, cuneus and the inferior-middle frontal gyrus. Neutral expressions activated the inferior occipital and fusiform gyri, cuneus, insula and the caudate. Happy expressions activated only the inferior-middle occipital and fusiform gyri and the cuneus (supplementary appendix 1; figure 1). In general, the magnitude of activations across all conditions in healthy participants was greater than in patients.

#### Depression and Brain Activity in Patients.

##### Regression Analyses 

**Fig. 2.**
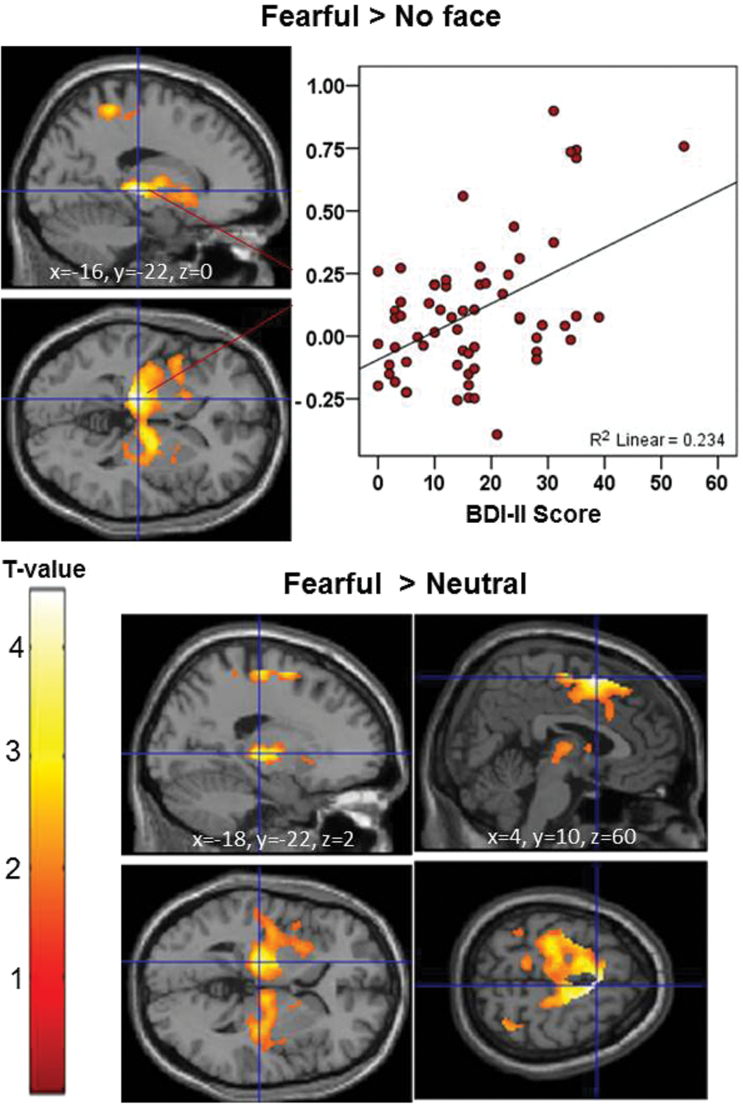
Brain activity positively associated with Beck Depression Inventory (BDI-II) depression with associated Montreal Neurological Institute (MNI) *x*, *y* and *z* coordinates (maps thresholded at *P* = .05 uncorrected; displayed clusters significant at corrected *P* ≤ .05). For each coordinate, sagittal and axial slices are shown. The left hemisphere is shown at the top of the axial slices.

Depression (BDI-II) scores correlated significantly positively with activity during fearful expressions (>no face) in the left thalamus extending to the para-post-pre-central gyrus, putamen-globus pallidus, supramarginal gyrus, insula and inferior-middle frontal gyrus (table 2, figure 2). BDI-II scores also correlated significantly positively with activity for fearful expressions when compared with neutral (fearful > neutral) expressions in the right SFG extending to middle/medial frontal, left precentral and left anterior cingulate gyri. Another cluster, very similar to the cluster found to correlate positively with BDI-II scores during fearful > nonface contrast, with near identical peak in the left thalamus, was also present at uncorrected significance (table 2, figure 2). BDI-II scores did not correlate significantly (positively or negatively) with activation of any area during angry, neutral or happy expressions.

Participant-specific activation values extracted from peak voxel of the above 3 clusters that associated positively with BDI-II scores (peak of cluster 1 in the thalamus, fearful > no face control contrast; of cluster 2 in the superior frontal gyrus, and of cluster 3 in the thalamus, both fearful > neutral face contrast) did not correlate significantly with PANSS total or subscale scores (all *r* values <.17). Participant-specific activation values associated positively with BDI-II scores also did not correlate with age (*r* values −.009 to .045; all *P* > .72), duration of illness (*r* values −.015 to .03; all *P* > .80) or antipsychotic medication dose (*r* values −.039 to −.025; all *P* > .76), and their correlation with BDI-II scores remained significant with near identical *P* values (all *P* ≤ .001) after controlling (partial *r*) for age, illness duration or doses of antipsychotic medication.

##### Categorical Analyses 

**Fig. 3.**
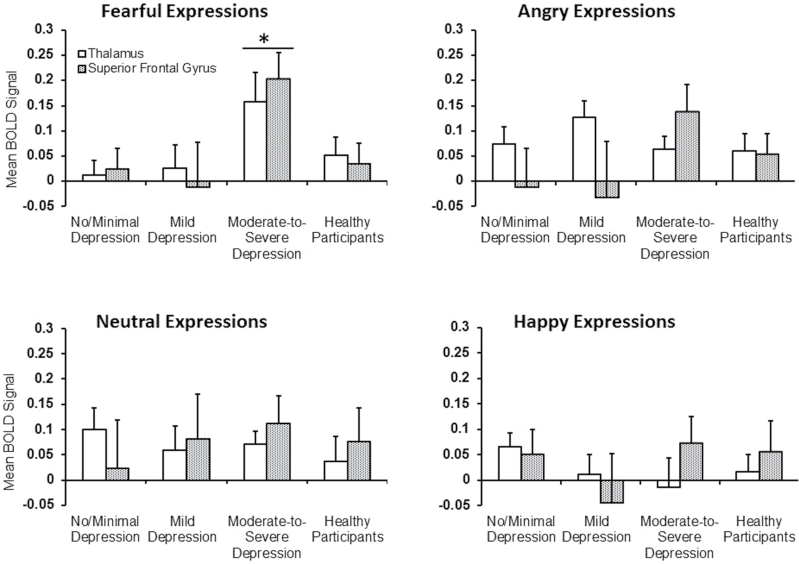
Mean functional magnetic resonance imaging (fMRI) response in the thalamic and superior frontal gyrus clusters (correlated with Beck Depression Inventory [BDI-II] scores during fearful, but not other, expressions) in patients with varying depression levels and healthy participants (*indicates *P* < .05; higher than all other groups).

The Region × Emotion type × Depression (2×4 × 3) ANOVA to examine differences between patient subgroups revealed a significant Region × Emotion type effect (*F*_3,180_ = 2.82, *P* = .04) and a trend for Emotion type × Depression effect (*F*_6,180_ = 1.76, *P* = .11) with a significant linear trend (*F*_2,603_ = 3.81, *P* = .026). Follow-up analyses of these effects using Region × Depression ANOVAs separately for fearful, angry, happy and neutral expressions revealed a significant Depression effect for fearful expressions (*F*_2,60_ = 4.86, *P* = .01). This effect was due to significantly higher activity across the thalamic and SFG clusters in moderate-to-severe depression subgroup, relative to no/minimal (*F*_1,44_ = 8.97, *P* = .004) and mild depression (*F*_1,36_ = 5.32, *P* = .03) subgroups; no/minimal and mild depression subgroups did not differ from each other (*F*_1,40_ = 0.04, *P* = .85; figure 3). Region × Depression ANOVAs (separately) for angry, happy and neutral expressions did not reveal any significant effects (figure 3).

Further Region × Group (no/minimal depression, mild depression and moderate-to-severe depression patients, and healthy participants) ANOVA to specifically probe activity differences for fearful expressions in moderate-to-severe depression patients, relative to healthy participants, also revealed a significant Group effect (*F*_3,79_ = 3.88, *P* = .01). This effect was due to significantly higher activity in the moderate-to-severe depression patient subgroup, relative to the healthy group (*F*_1,39_ = 5.89, *P* = .02) and no/minimal and mild depression patient subgroups (effects already described). Patients with no/minimal (*F*_1,43_ = 0.36, *P* = .55) or mild depression (*F*_1,35_ = 0.34, *P* = .56) did not differ from healthy participants (or each other, as described earlier).

## Discussion

Our primary aim was to determine the brain activity associated with depression, as indexed with BDI-II, in patients with chronic schizophrenia during implicit processing of facial affect. The findings partially confirmed our a priori hypothesis in that there was a significantly positive association between depression (BDI-II) scores and brain activity to facial expressions of fear. This association was robustly present (fearful > no face control) in a cluster that peaked in the left thalamus and extended to include parts of the insula, globus pallidus, and inferior-middle frontal and pre-post-para-precentral gyri. Although the thalamic cluster was present at the uncorrected level in fearful > neutral expressions contrast, it was in the same location as detected with fear > no face control contrast, and extended to include parts of the caudate, putamen, insula and hippocampus. In addition, during fearful > neutral contrast, bilateral activity in the superior-middle-medial frontal gyrus, extending to the precentral gyrus and anterior cingulate, was associated significantly positively with BDI-II scores. Importantly, patients with moderate-to-severe depression had significantly greater brain activity in these regions during fearful expressions, relative to patients with no/minimal/mild depression and healthy participants.

In general, our fMRI observations fit with previous findings of enhanced brain activity seen in MDD patients in response to facial expressions of fear.^15,43^ However, unlike previous findings in MDD which often show a hyperactive amygdala response to fearful expressions,^16–18^ we did not find amygdala activation to associate with depression in psychosis patients, more likely because of their long-term antipsychotic use^44–46^ or other diagnosis-related issues,^24,43^ rather than amygdala response habituation with repeated exposure to facial stimuli during the practice and fMRI sessions,^47^ since there was amygdala activation to fearful expressions in healthy participants (figure 1) with identical task and scanning parameters. Patients with moderate-to-severe depression in this study also showed the strongest activity in the regions that showed “fear expressions-depression relationship” but were not significantly different from patients with no/minimum/mild depression or healthy participants during angry expressions. Importantly, Gur and colleagues^25^ also reported enhanced brain response to fearful, but not angry, expressions to be significantly associated with a high severity of flat affect in schizophrenia (*n* = 16) and this association was present in the thalamus and hippocampus in addition to the amygdala. It is also relevant in this context that we^32^ previously found a more robust reduction, following cognitive behavior therapy for psychosis (CBTp), in activity of the inferior frontal, insula, thalamus and putamen areas during fearful, than during angry, expressions; and improvements in subjective depression are a consistent and important outcome of CBTp.^48,49^ Taken together, these finding indicate a consistent relationship between depression and altered neural response to fearful expressions, a signal of uncertain threat,^50^ in schizophrenia. Interestingly, there is recent meta-analytic evidence that schizophrenia patients (as a group) show under-activation in the entire facial emotion processing network whereas those with bipolar disorder (BD) show greater thalamic engagement during facial emotion processing.^24^ The present findings regarding depression in the context of psychosis, therefore, seem related to emotion processing abnormalities in BD, and may point towards the use of therapies known to be effective for treating depressed mood in BD for treating depression in schizophrenia.

Of the regions that showed a positive association with depression during the viewing of fearful facial expressions, the pulvinar thalamus, with projections to the amygdala,^51^ has been strongly implicated in the control of visual attention,^52–54^ specifically during rapid detection of threatening stimuli.^55,56^ A negative correlation between avoidance response to threatening stimuli and activation of the pulvinar thalamus, insula and striatum has also been previously demonstrated in healthy humans.^57^ It is thus possible that patients with mild/severe depression attended fearful expressions to a greater extent and possibly engaged in explicit processing of threat-related information.^58^

The strengths of this study include the use of a large sample to examine depression-facial affect processing relationship, ie, sufficient power to investigate the questions of interest. The main limitation of this study is that it is cross-sectional in nature and therefore there is no direct evidence of cause and effect. Further limitations include a lack of reliable information on the proportion of patients who met DSM criteria for current/past MDD and possible confounding effects of different types and dosages of medications. It would be valuable in future to conduct longitudinal investigations while monitoring depression and medications, and to directly compare patients with schizophrenia and varying levels of depression to MDD and BD patients with varying levels of psychotic symptoms in their responses to facial affect, relative to an appropriate baseline (eg, scrambled face)^59^ within the same study.

In conclusion, this study provides the first evidence that psychosis patients with a high level of subjective depression show enhanced brain response to fearful facial expressions, particularly in the thalamus. Our findings suggest an overlap between schizophrenia patients with moderate-to-severe depression and BD patients, the latter of which reliably show greater thalamic involvement during facial affect processing. Unlike previous findings in MDD,^16–18^ we found no evidence of amygdala activation during fearful expression to associate with depression in the context of psychosis.

## Supplementary Material

Supplementary material is available at http://schizophreniabulletin.oxfordjournals.org.

## Funding

This research was supported by the Wellcome Trust (067427/z/02/z). V.K. is part funded by the Biomedical Research Centre for Mental Health at the Institute of Psychiatry, King’s College London, and the South London and Maudsley NHS Foundation Trust, UK.

## Supplementary Material

Supplementary Data
