# Supplementary material for: Mapping Depression in Schizophrenia: A Functional Magnetic Resonance Imaging Study
Source: Schizophr Bull. 2015 Dec 27;42(3):802–13. doi: 10.1093/schbul/sbv186 (PMC4838102; doi:10.1093/schbul/sbv186)
Supplement: Supplementary Data [file supp_42_3_802__index.html]

Mapping Depression in Schizophrenia: A Functional Magnetic Resonance Imaging Study — Mapping Depression in Schizophrenia: A Functional Magnetic Resonance Imaging Study — Supplementary Data 

# Mapping Depression in Schizophrenia: A Functional Magnetic Resonance Imaging Study

## Supplementary Data

Data files

- Supplementary Data - Supplementary Data
