# Supplementary material for: Mapping Depression in Schizophrenia: A Functional Magnetic Resonance Imaging Study
Source: Schizophr Bull. 2015 Dec 27;42(3):802–13. doi: 10.1093/schbul/sbv186 (PMC4838102; doi:10.1093/schbul/sbv186)
Supplement: Supplementary Data [file supp_sbv186_Appendix_1_19_10_2015.doc]

Appendix 1. Activation increases in patients and healthy participants to emotive and neutral facial expressions compared to no face control stimuli (height threshold *p<*.005 uncorrected).

| **Patients** | | | | | | | | |
| --- | --- | --- | --- | --- | --- | --- | --- | --- |
| Brain Area | BA | Voxels  (n) | Side | MNI Coordinates | | | Voxel T | Cluster-corrected *p* |
|  |  |  |  | x | y | z |  |  |
| **Fearful** | | | | | | | | |
| Inferior frontal gyrus | 9 | 911 | Right | 48 | 8 | 32 | 6.29 | .015 |
|  | 46 |  | Right | 48 | 34 | 10 | 3.30 |  |
|  | 46 |  | Right | 44 | 28 | 16 | 3.25 |  |
| **Angry** | | | | | | | | |
| Inferior frontal gyrus | 9 | 1788 | Left | -46 | 14 | 24 | 7.95 | .001 |
|  | 45 |  | Left | -50 | 24 | 20 | 5.63 |  |
|  | 45 |  | Left | -44 | 32 | 4 | 4.27 |  |
|  | 9 | 2227 | Right | 52 | 16 | 26 | 7.87 | .000 |
|  | 9 |  | Right | 46 | 10 | 26 | 7.63 |  |
|  | 46 |  | Right | 48 | 34 | 14 | 6.22 |  |
| Superior parietal lobule | 7 | 1060 | Right | 26 | -62 | 44 | 5.45 | .011 |
|  | 7 |  | Right | 30 | -70 | 32 | 5.27 |  |
| Middle occipital gyrus | 19 |  | Right | 34 | -78 | 14 | 4.28 |  |
| Ventral posterior medial nucleus (thalamus) |  | 858 | Left | -16 | -20 | 4 | 4.93 | .026 |
| Globus Pallidus |  |  | Left | -22 | -12 | -4 | 4.46 |  |
| Thalamus |  |  | Left | -26 | -30 | 4 | 4.31 |  |
| Parahippocampal gyrus | 27 | 817 | Right | 26 | -30 | -4 | 4.39 | .032 |
|  | 34 |  | Right | 24 | 0 | -16 | 4.34 |  |
| Thalamus |  |  | Right | 8 | -26 | -2 | 4.06 |  |
| **Neutral** | | | | | | | | |
| Claustrum |  | 3419 | Right | 32 | -4 | 0 | 5.74 | .000 |
| Thalamus |  |  | Right | 18 | -32 | -2 | 5.48 |  |
| Middle frontal gyrus | 9 |  | Right | 50 | 16 | 30 | 4.89 |  |
|  | 9 | 3593 | Left | -40 | 8 | 32 | 5.58 | .000 |
| Precentral gyrus | 6 |  | Left | -40 | -12 | 58 | 4.98 |  |
|  | 4 |  | Left | -36 | -20 | 62 | 4.93 |  |
| Putamen |  | 1769 | Left | -30 | -18 | -2 | 4.94 | .001 |
| Globus pallidus |  |  | Left | -20 | -4 | -8 | 4.03 |  |
| Thalamus |  |  | Left | -12 | -16 | 6 | 3.69 |  |
| Middle frontal gyrus | 6 |  | Left | -18 | -14 | 64 | 3.86 |  |
| Postcentral gyrus | 3 |  | Left | -30 | -34 | 66 | 3.75 |  |
| **Happy** | | | | | | | | |
| None significant | | | | | | | | |
| **Healthy Participants** | | | | | | | | |
| **Fearful** | | | | | | | | |
| Inferior occipital gyrus | 19 | 2849 | Right | 32 | -90 | -10 | 10.72 | < .001 |
| Cuneus | 18 |  | Right | 24 | -100 | -6 | 10.72 |  |
| Inferior occipital gyrus | 19 |  | Right | 46 | -80 | -10 | 9.42 |  |
| Cuneus | 18 | 2000 | Left | -26 | -100 | -4 | 9.15 | < .001 |
| Middle occipital gyrus | 18 |  | Left | -38 | -88 | -4 | 6.51 |  |
| Cerebellum |  |  | Left | -34 | -78 | -22 | 5.83 |  |
| Middle frontal gyrus | 46 | 805 | Right | 52 | 40 | 22 | 5.98 | .017 |
| Inferior frontal gyrus | 9 |  | Right | 42 | 6 | 26 | 4.92 |  |
| Middle frontal gyrus | 46 |  | Right | 42 | 18 | 24 | 4.44 |  |
| Globus pallidus |  | 1507 | Left | -26 | -18 | -2 | 5.40 | < .001 |
| Putamen extends into parahippocampal gyrus and amygdala |  |  | Left | -26 | -2 | -8 | 4.99 |  |
| Insula | 13 |  | Left | -40 | -20 | 8 | 4.98 |  |
| Parahippocampal gyrus extends into amygdala | 28 | 802 | Right | 22 | -16 | -16 | 4.48 | .017 |
| Hippocampus |  |  | Right | 30 | -22 | -12 | 4.47 |  |
| Medial geniculate (thalamus) |  |  | Right | 16 | -26 | -8 | 4.27 |  |
| **Angry** | | | | | | | | |
| Inferior occipital gyrus | 18 | 2700 | Right | 40 | -86 | -14 | 12.28 | < .001 |
| Lingual gyrus | 18 |  | Right | 24 | -98 | -8 | 11.97 |  |
| Inferior occipital gyrus | 18 |  | Right | 34 | -90 | -8 | 11.41 |  |
| Cuneus | 18 | 1313 | Left | -26 | -100 | -4 | 9.50 | .003 |
| Culmen |  |  | Left | -38 | -50 | -28 | 5.94 |  |
| Inferior occipital gyrus | 18 |  | Left | -32 | -84 | -10 | 5.67 |  |
| Inferior-middle frontal gyrus | 45 | 905 | Right | 48 | 18 | 22 | 4.86 | .011 |
|  | 9 |  | Right | 46 | 6 | 30 | 4.83 |  |
| **Neutral** | | | | | | | | |
| Inferior occipital gyrus | 17 | 3388 | Right | 28 | -96 | -8 | 13.67 | < .001 |
|  | 19 |  | Right | 40 | -86 | -12 | 13.47 |  |
|  | 18 |  | Right | 32 | -90 | -12 | 12.24 |  |
| Cuneus | 18 | 1924 | Left | -24 | -102 | -4 | 9.83 | < .001 |
|  | 17 |  | Left | -20 | -94 | -10 | 8.69 |  |
| Fusiform gyrus | 19 |  | Left | -36 | -74 | -14 | 7.33 |  |
| Insula | 13 | 2307 | Right | 32 | 22 | 14 | 6.75 | < .001 |
| Caudate |  |  | Right | 16 | 0 | 14 | 4.77 |  |
| Inferior frontal gyrus | 45 |  | Right | 54 | 26 | 24 | 4.40 |  |
| **Happy** | | | | | | | | |
| Cuneus | 18 | 2073 | Right | 26 | -98 | -6 | 12.48 | .001 |
| Middle-inferior occipital gyrus | 18 |  | Right | 32 | -92 | -6 | 12.36 |  |
|  | 18 |  | Right | 36 | -88 | -12 | 9.94 |  |
|  | 18 | 1340 | Left | -34 | -90 | -4 | 7.64 | .013 |
| Cuneus | 18 |  | Left | -24 | -102 | -4 | 7.41 |  |
| Fusiform gyrus | 19 |  | Left | -42 | -72 | -16 | 6.20 |  |

BA: Brodmann area; MNI: Montreal Neurological Institute.
